# Triptolide treatment reduces Alzheimer’s disease (AD)-like pathology through inhibition of BACE1 in a transgenic mouse model of AD

**DOI:** 10.1242/dmm.018218

**Published:** 2014-12

**Authors:** Qi Wang, Bing Xiao, Shuqin Cui, Hailong Song, Yanjing Qian, Lin Dong, Haiting An, Yanqiu Cui, Wenjing Zhang, Yi He, Jianliang Zhang, Jian Yang, Feilong Zhang, Guanzheng Hu, Xiaoli Gong, Zhen Yan, Yan Zheng, Xiaomin Wang

**Affiliations:** 1 Department of Physiology, Department of Neurobiology, Key Laboratory for Neurodegenerative Disorders of the Ministry of Education, Capital Medical University, Beijing 100069, PR China.; 2 Department of Medicine, Dezhou University, Dezhou 253023, PR China.; 3 Capital Medical University Yanjing Medical College, Beijing 101300, PR China.; 4 Beijing Institute for Brain Disorders, Beijing 100069, PR China.; 5 Department of Physiology and Biophysics, State University of New York at Buffalo, Buffalo, NY 14214, USA.

**Keywords:** Alzheimer’s disease, Amyloid β, 5XFAD mice, BACE1, Inflammation, Triptolide

## Abstract

The complex pathogenesis of Alzheimer’s disease (AD) involves multiple contributing factors, including amyloid β (Aβ) peptide accumulation, inflammation and oxidative stress. Effective therapeutic strategies for AD are still urgently needed. Triptolide is the major active compound extracted from *Tripterygium wilfordii* Hook.f., a traditional Chinese medicinal herb that is commonly used to treat inflammatory diseases. The 5-month-old 5XFAD mice, which carry five familial AD mutations in the β-amyloid precursor protein (*APP*) and presenilin-1 (*PS1*) genes, were treated with triptolide for 8 weeks. We observed enhanced spatial learning performances, and attenuated Aβ production and deposition in the brain. Triptolide also inhibited the processing of amyloidogenic APP, as well as the expression of βAPP-cleaving enzyme-1 (BACE1) both *in vivo* and *in vitro*. In addition, triptolide exerted anti-inflammatory and anti-oxidative effects on the transgenic mouse brain. Triptolide therefore confers protection against the effects of AD in our mouse model and is emerging as a promising therapeutic candidate drug for AD.

## INTRODUCTION

Alzheimer’s disease (AD) is the most common neurodegenerative disease and constitutes approximately two-thirds of all cases of dementia (Reitz et al., 2011). It progressively impairs memory, judgment, decision-making and language (Nussbaum and Ellis, 2003; Goedert and Spillantini, 2006). The etiology and pathogenesis of the disease are still not well understood. The pathological hallmarks of AD include extracellular senile plaques, which are composed mainly of 40- to 42-amino-acid amyloid β (Aβ) peptides, and intracellular neurofibrillary tangles. These hallmarks are most evident in the cortex and the hippocampus (Braak and Braak, 1991; Selkoe, 1993). Aβ is thought to play an important role in AD through triggering a series of processes called the amyloid cascade that eventually lead to dementia (Hardy and Selkoe, 2002; Huang and Mucke, 2012; Mucke and Selkoe, 2012). Inhibition of the production of Aβ is therefore considered to be a potential therapeutic strategy for the treatment of AD (Mangialasche et al., 2010). In addition to Aβ toxicity, inflammation and oxidative stress are also important during the development of AD (Ballard et al., 2011). AD is therefore a complex aging-related disease that can be caused by a variety of genetic and environmental factors.

There are currently four therapeutic agents for AD that have been approved by the US Food and Drug Administration (FDA) (donepezil, rivastigmine, galantamine and memantine), which are unable to prevent or reverse the disease progression and are only modestly efficacious. Therefore, other more effective treatments are urgently needed. Although the majority of agents that exclusively target amyloid-related alterations have so far been unsuccessful (Corbett et al., 2012), this is likely to be due to insufficient knowledge of single molecular targets and because late interventions are ineffective.

Triptolide is a lipophilic extract and the major active compound isolated from Tripterygium wilfordii Hook.f., a traditional Chinese medicinal herb that has been used to treat inflammatory diseases for centuries. Our laboratory and others have previously reported that triptolide exerts multiple biological activities in various types of brain cells (Zheng et al., 2013), including the inhibition of microglial activation and the release of pro-inflammatory factors (Zhou et al., 2003; Zhou et al., 2005; Jiao et al., 2008; Lee et al., 2012), alleviating the inflammatory response and promoting the synthesis and release of nerve growth factor (NGF) in astrocytes (Li et al., 2003; Li et al., 2004a; Xue et al., 2007). Furthermore, triptolide directly prevents Aβ-induced neuronal apoptosis and CXCL1-induced cytotoxicity (Wang et al., 2013b), as well as promoting the synaptophysin expression of hippocampal neurons in the AD cellular model (Nie et al., 2012). This suggests that triptolide exerts protective roles in the AD brain through multiple pharmacological mechanisms. However, it has not yet been elucidated whether triptolide has therapeutic effects in AD models *in vivo*.

In this study, β-amyloid precursor protein (APP) and presenilin-1 (PS1) double-transgenic mice, which co-express five familial AD mutations (5XFAD) and rapidly recapitulate major features of AD pathology, were used to evaluate the therapeutic effects and mechanisms of action of triptolide on AD. We found that treatment with triptolide improved the spatial learning performance of the AD transgenic mice and attenuated the production and accumulation of Aβ in the hippocampus and cortex. We also observed a decrease in the expression of β-secretase βAPP-cleaving enzyme-1 (BACE1), the rate-limiting enzyme that catalyzes Aβ production, and increased levels of neprilysin (NEP), a key enzyme that degrades Aβ. Importantly, we also identified that amyloidogenic APP processing is inhibited by treatment with triptolide, both *in vivo* and *in vitro*. Finally, triptolide exerted anti-inflammatory and anti-oxidative effects on the brains of the transgenic mice. This study suggests that triptolide has potential as a novel therapeutic agent that targets multiple aspects of AD.

**TRANSLATIONAL IMPACT**

**Clinical issue**

Alzheimer’s disease (AD) is one of the most common neurodegenerative diseases, and accounts for approximately two-thirds of all cases of dementia. It progressively impairs memory, judgment, decision-making and language. The etiology and pathogenesis of the disease are still not well understood. There are currently four FDA-approved therapeutic agents against AD – donepezil, rivastigmine, galantamine and memantine – which, however, are unable to prevent or reverse the disease progression, and are only modestly efficacious. Although β amyloid (Aβ) is thought to play an important role in AD, by triggering a series of processes called the amyloid cascade that finally lead to dementia, the majority of agents that exclusively target amyloid-related alterations have so far been unsuccessful.

**Results**

In this study, Wang et al. provide evidence that triptolide, an active compound extracted from the traditional Chinese herb *Tripterygium wilfordii*, improved spatial learning and reduced AD-related pathological hallmarks (including amyloid plaque deposition) in an AD mouse model that co-expresses five familial AD mutations (5XFAD). Authors report that 8 weeks of treatment with triptolide in five-month-old 5XFAD mice decreased the aggregation of Aβ in the brain by altering the processing of its precursor protein (amyloid precursor protein, APP). Furthermore, triptolide suppressed glial activation and the inflammatory response, and additionally reduced oxidative stress in these animals, all factors that are known to be associated with AD.

**Implications and future directions**

This study suggests that triptolide might be a potential therapeutic agent for targeting multiple aspects of AD. Insufficient knowledge of the molecular pathways upstream of Aβ accumulation, of single-target strategies and of late interventions are considered as the bottlenecks impeding the road to translate agents that exclusively inhibit Aβ accumulation into clinical application. Given its multi-target action, triptolide might represent a new treatment solution for AD and also for some AD-related pathologies.

## RESULTS

### Triptolide treatment improves the spatial learning of 5XFAD mice

**Fig. 1.**
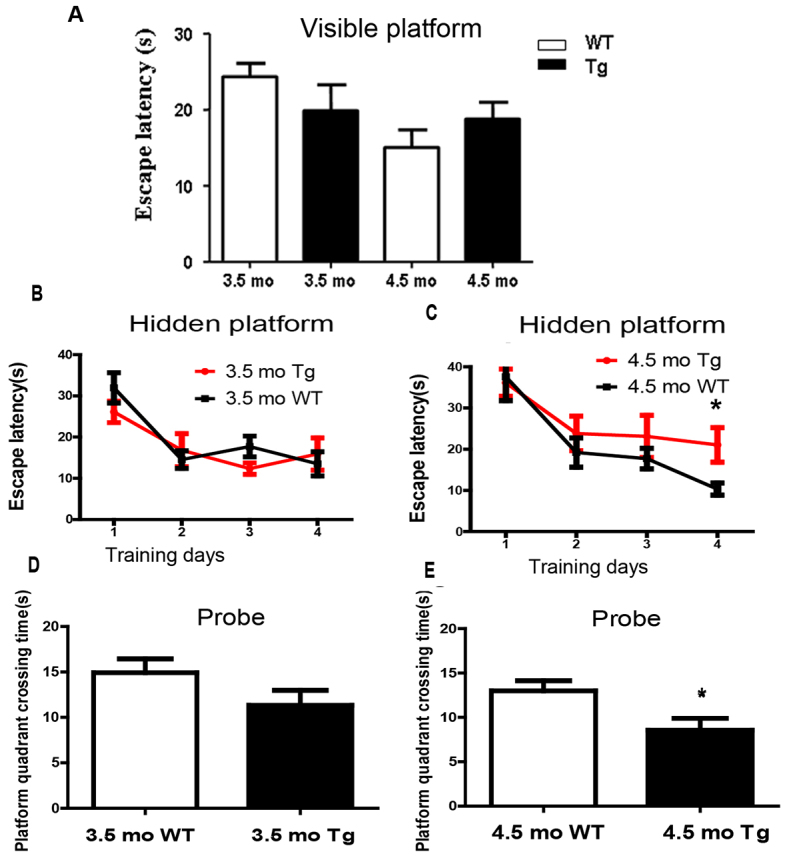
**The spatial reference learning and memory of 5XFAD mice was impaired at the age of 4.5 months.** (A) The 5XFAD mice (Tg) and wild-type mice (WT) were subjected to visible platform training. (B,C) The average escape latency (mean±s.e.m.) to reach the hidden platform was used to evaluate the learning performance of 5XFAD mice and wild-type mice at 3.5 months or 4.5 months (mo) of age. (D,E) A probe test after 4 days of training was performed to examine the function of memory retention of 5XFAD mice and wild-type mice. The time crossing in the platform quadrant was assessed. Data represent mean±s.e.m.; *n*≥10 animals per group. *P<0.05 versus WT, Student’s *t*-test within each age group.

To assess the spatial learning and memory of untreated 5XFAD mice, we subjected 3.5- to 4.5-month-old transgenic mice and their wild-type littermates to a Morris water maze test. In the visible platform task, 5XFAD transgenic mice aged 3.5 or 4.5 months showed a similar ability to find the platform as their corresponding wild-type controls (Fig. 1A). This suggested that there was no difference in the ability of the transgenic or wild-type mice to swim or visually discriminate. The 3.5-month-old transgenic mice showed no significant difference in their learning ability in order to locate the hidden platform in the spatial reference memory test compared with wild-type controls (Fig. 1B). In contrast, the 4.5-month-old transgenic mice were less efficient at finding the hidden platform (Fig. 1C). Comparison of swimming speeds during the hidden platform training also revealed no significant differences between the transgenic and control mice (data not shown). To evaluate spatial memory retention, the platform was removed for a probe trial 24 hours after the last day of hidden platform training. The 4.5-month-old, but not the 3.5-month-old, 5XFAD mice spent significantly less time searching in the platform quadrant than wild-type controls (Fig. 1D,E).

**Fig. 2.**
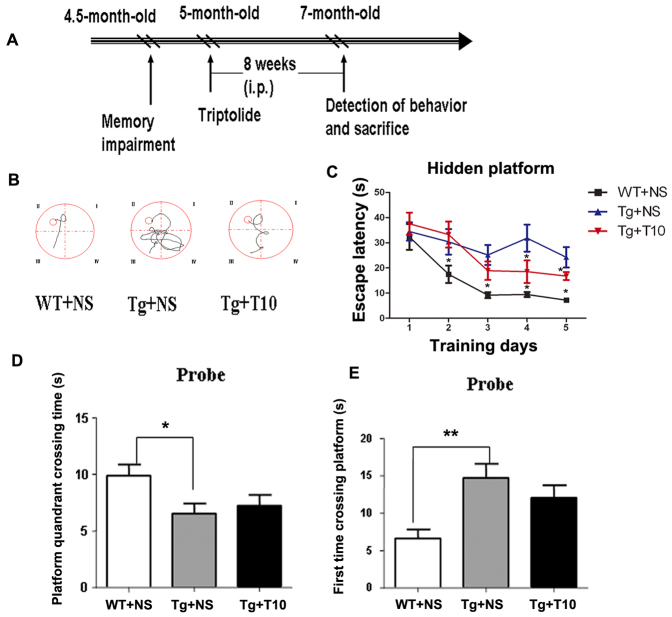
**Chronic administration of triptolide improved the spatial memory of 5XFAD mice in the Morris water maze.** (A) Treatment schedule for the experiment. i.p., intraperitoneal. (B) The escape strategy during the hidden platform task adopted by saline-treated wild-type mice (WT+NS), saline-treated 5XFAD mice (Tg+NS) and triptolide (T10)-treated 5XFAD mice (Tg+T10) was detected by using a camera. (C) The escape latency of these three groups of mice to find the hidden platform was recorded on every training day. (D,E) One day after finishing the acquisition task, a probe trial was performed to evaluate spatial memory. The platform quadrant crossing time (D), and the time required for the first crossing over the platform site (E), were used to determine the memory retention of these three groups of mice. All values represent mean±s.e.m.; *n*=8–10 animals per group. *P<0.05, **P<0.01 versus Tg+NS, samples were compared using one-way ANOVA with Tukey’s *post hoc* test. NS, normal saline.

According to the behavioral phenotype, we selected 5-month-old 5XFAD mice, which had shown deficits in learning and memory, to assess the therapeutic effects of triptolide (20 μg/kg of body weight, treated for 8 weeks) on the symptomatic AD model. The behavioral performance of mice was determined at 7 months old (Fig. 2A). As shown in Fig. 2B, the escape pathway on the fifth day of training that was adopted by the 5XFAD mice treated with saline in the hidden platform task was more complicated and longer than that used by control mice treated with saline. Importantly, the behavioral impairment could be rescued upon treatment with triptolide. The escape latency of 5XFAD mice to find the hidden platform was longer than that of controls on day 4 and 5 of training, whereas the triptolide-treated 5XFAD mice demonstrated an improved learning ability in the spatial learning task compared with saline-treated 5XFAD mice (Fig. 2C). However, compared with wild-type control mice, both saline- and triptolide-treated 5XFAD mice spent significantly less time in the target quadrant (Fig. 2D) and took more time to find the quadrant that had originally contained the platform (Fig. 2E) during the probe trial, which was performed 24 hours after the last training session in the hidden platform task. This suggests that triptolide prevented the deterioration of learning ability but did not improve the memory retention of 5XFAD mice. Meanwhile, we also monitored the side effects of triptolide (20 μg/kg of body weight intraperitoneally for 8 weeks) on peripheral tissues of these mice and did not find any toxicity in a wide variety of tissues, including the heart, liver, spleen, lung and kidney (supplementary material Fig. S2). Moreover, treatment with triptolide did not affect the body weight of the 5XFAD mice (supplementary material Fig. S3).

### Triptolide treatment attenuates the Aβ burden and inhibits Aβ generation in 5XFAD brains

To detect the formation of amyloid plaques in the brains of transgenic mice and to determine the extent of the pathological changes that occurred after treatment with triptolide, brain sections were stained with the Aβ-specific antibody 6E10. In the brains of 5-month-old untreated 5XFAD mice, amyloid plaques were distributed predominantly on deep cortical layers, with fewer deposits observed in the hippocampus, as shown in supplementary material Fig. S4A. However, by the age of 7 months, the plaques had spread to fill most of cortex and hippocampus. To confirm these findings, ELISA was used to quantify the soluble and insoluble forms of Aβ in the hippocampus. Both forms of the 42-amino-acid Aβ peptide (Aβ42) increased significantly with age between 5 and 7 months (supplementary material Fig. S4B,C). By contrast, only the soluble form of the 40-amino-acid Aβ peptide (Aβ40) increased with age in the brains of transgenic mice (supplementary material Fig. S4D), and no changes in the insoluble form were observed (supplementary material Fig. S4E). These results are consistent with previous observations that Aβ42 deposits in 5XFAD mice rapidly increased with age, but Aβ40 levels were lower in amyloid deposits (Oakley et al., 2006).

**Fig. 3.**
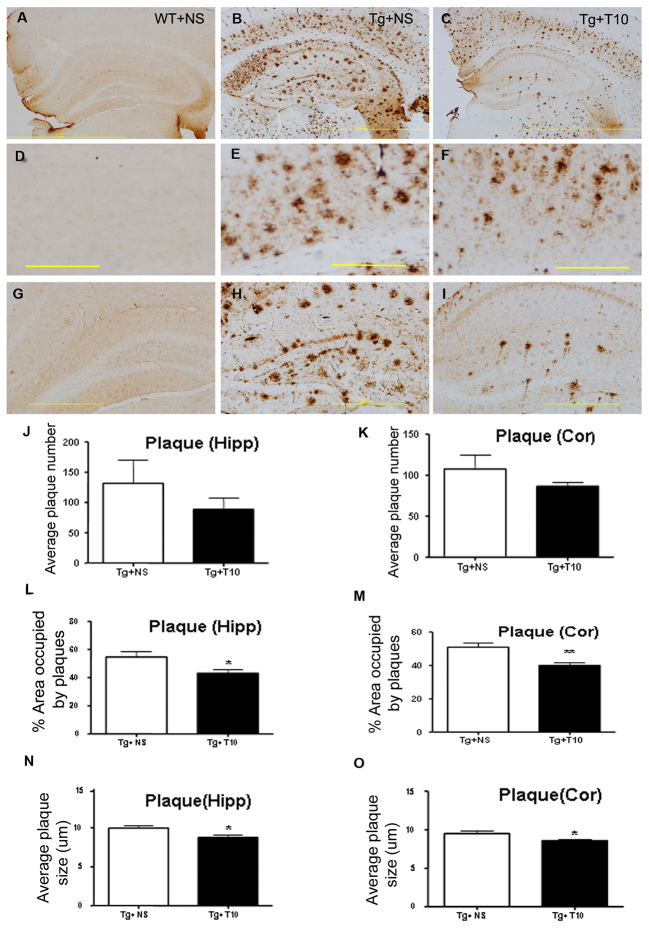
**Treatment with triptolide attenuated the formation of Aβ-enriched plaques in the brains of 5XFAD mice.** (A–I) Immunoreactive Aβ plaques were detected by staining with the specific 6E10 antibody. No positive staining was detected in the brains of wild-type control mice (WT+NS) (A,D,G), and plaques were detected in both the cortex (E) and the hippocampus (H) of saline-treated 5XFAD brains (Tg+NS) (B) and triptolide (T10)-treated 5XFAD mice (Tg + T10) (C,F,I). (J–O) The quantification of the total number of plaques (J,K), the percentage area occupied by plaques (L,M), the average plaque size (N,O) in the cortex (Cor) or hippocampus (Hipp) of Tg+NS and Tg+T10 groups of mice were analyzed by using Image Pro Plus 6.0 software. All values are presented as means±s.e.m.; *n*=6 animals per group. *P<0.05, **P<0.01, Student’s *t*-test. NS, normal saline. Scale bars: 2 mm (A–C); 500 μm (D–I).

To investigate whether the administration of triptolide could reduce the levels of Aβ, we used immunohistochemistry to analyze the brain sections of 5XFAD mice that had been treated with triptolide for 8 weeks (Fig. 3A–I). Although the number of plaques in both the cortex and hippocampus of triptolide-treated 5XFAD mice was comparable to the saline-treated 5XFAD mice (Fig. 3J,K), the average area occupied by plaques in the triptolide-treated 5XFAD mice was reduced by ~20% and 17% of that of saline-treated 5XFAD mice in the cortex and hippocampus, respectively (Fig. 3L,M), whereas the average plaque size (μm) in triptolide-treated 5XFAD mice was also decreased significantly in comparison with that of saline-treated 5XFAD mice (Fig. 3N,O).

**Fig. 4.**
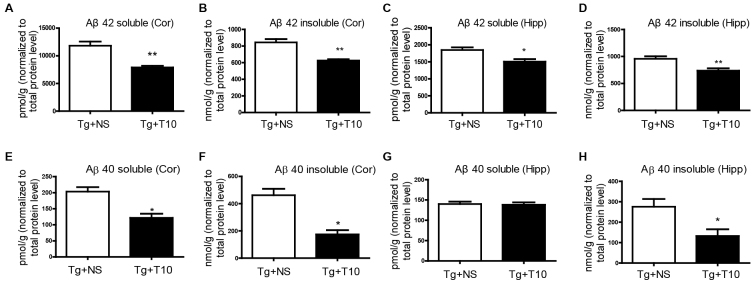
**Treatment with triptolide reduced Aβ production in the brains of 5XFAD mice.** (A–D) ELISA analyses revealed that treatment with triptolide (T10) induced a significant decrease in the levels of both soluble and insoluble Aβ42 in the cortex (Cor) and hippocampus (Hipp) of 5XFAD (Tg) mice. (E–H) Triptolide treatment reduced both soluble and insoluble Aβ40 levels in the cortex and the insoluble form of it in the hippocampus. NS, normal saline. All values are means±s.e.m.; *n*=6 animals per group. *P<0.05, **P<0.01, Student’s *t*-test.

We questioned whether the reduced plaque size was due to decreased Aβ concentrations in the brain. A sandwich ELISA assay was used to measure the levels of soluble and insoluble Aβ42 and Aβ40 in the cortex and hippocampus of transgenic mice that had been treated with triptolide or saline (Fig. 4A–H). After treatment with triptolide for 8 weeks, the levels of both soluble and insoluble Aβ42 were reduced by ~20% in the cortex and 30% in the hippocampus compared with those of saline-injected 5XFAD mice. In addition, the contents of Aβ40, except for soluble form of it, in the hippocampus of brains of 5XFAD mice that had been treated with triptolide was significantly reduced in comparison with saline-treated transgenic mice. These data suggest that triptolide can reduce the accumulation of Aβ and attenuate its burden in the AD brain.

### Triptolide administration does not influence the levels of exogenous human APP695 protein but downregulates β-amyloidosis of APP processing and upregulates NEP expression

To exclude the possibility that the decreased Aβ level seen in triptolide-treated mice was caused by altered APP levels, we assessed the expression of *APP695*, the exogenous human gene driven by the *Thy1* promoter, in the hippocampus and cortex. As shown in supplementary material Fig. S5A,B, no APP695 protein was detected by western blotting analysis in the brains of wild-type mice, but high levels of expression were seen in the hippocampus and cortex of brains from 5XFAD mice. Importantly, no significant difference was detected between saline- and triptolide-treated 5XFAD mice. This suggests that triptolide had no effect on APP expression in the brains of 5XFAD mice.

**Fig. 5.**
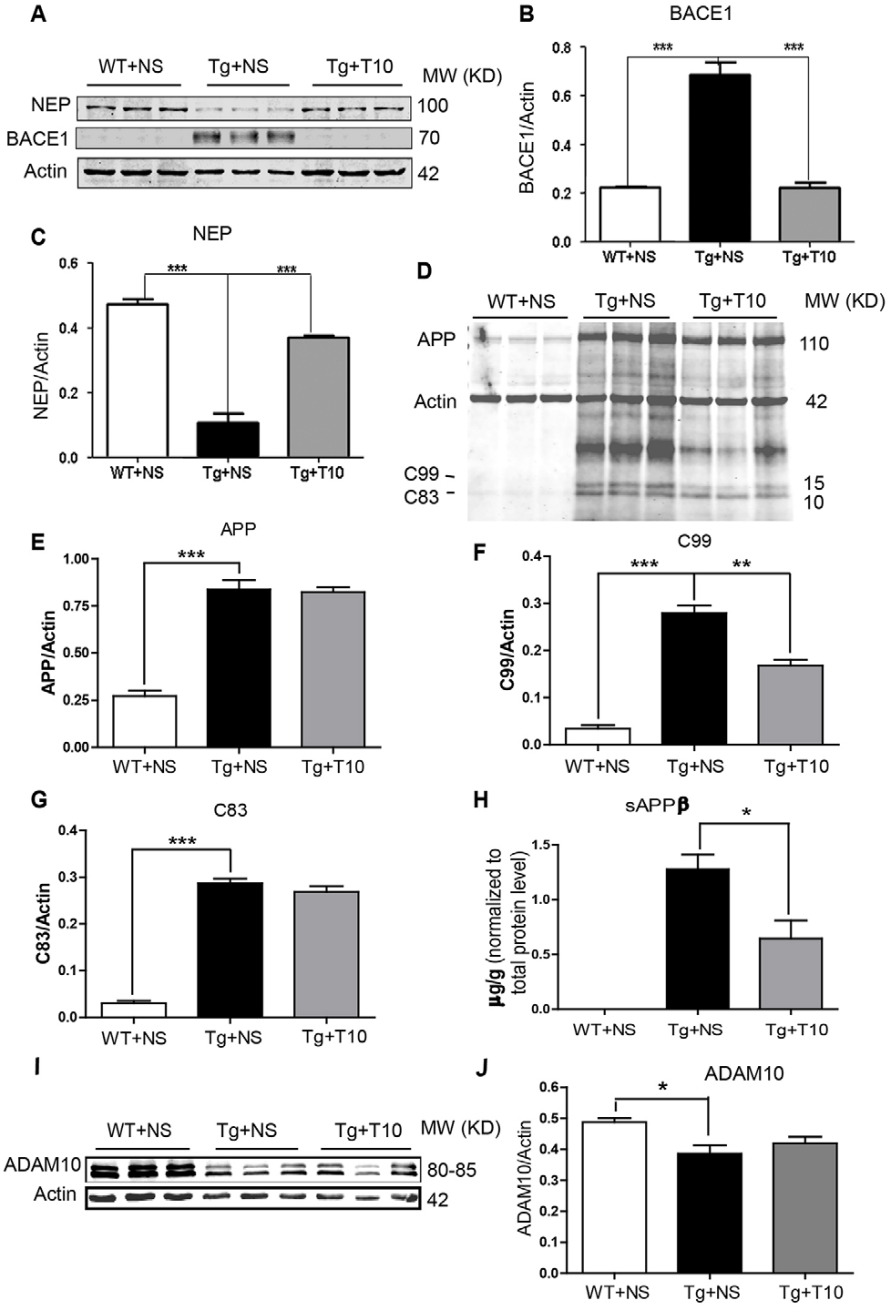
**Administration of triptolide modulated APP processing.** (A) The expression of NEP and BACE1 in the cortex was assessed by western blotting, and β-actin was used as an internal loading control. (B,C) Quantitative analysis of the expression of BACE1 and NEP in the cortex of wild-type (WT) and 5XFAD (Tg) mice treated with saline (NS) or triptolide (T10). (D) Full gel western blot result of APP and C99/C83 expression in the cortex of saline-treated wild-type mice (WT+NS), saline-treated 5XFAD mice (Tg+NS) and triptolide (T10)-treated 5XFAD mice (Tg+T10). Actin is used as a loading control. A rabbit polyclonal antibody against the APP C-terminal is used to recognize both APP and the C99 and C83 fragments. (E–G) Statistical analysis of APP, C99 and C83 levels normalized to that of actin. (H) Human soluble APPβ (sAPPβ) in the cortex of these three groups of mice was detected by a human sAPPβ ELISA kit. The result is shown as sAPPβ content normalized to the total protein level in the brain tissue. (I) The western blot shows the expression of ADAM10 in the cortex of these three groups of mice. (J) Quantitative data show the level of ADAM10 normalized to actin in the cortex of these three groups of mice. Values are means±s.e.m.; *n*=6 animals per group. *P<0.05, **P<0.01, ***P<0.001 versus Tg+NS, one-way ANOVA with Tukey’s *post hoc* test.

We next investigated whether the decrease in Aβ burden in the brains of 5XFAD mice that had been treated with triptolide was mediated by decreased Aβ production or increased Aβ clearance. We assessed the expression of BACE1 and NEP, and found that BACE1 protein levels in the cortex of 5XFAD mice were increased significantly compared with those of wild-type mice and were dramatically downregulated upon treatment with triptolide for 8 weeks (Fig. 5A,B). Similar results were obtained in the hippocampus (data not shown). In contrast, expression of NEP, a degradation enzyme for Aβ, was decreased in transgenic brains and upregulated upon triptolide treatment (Fig. 5A,C).

BACE1 is the rate-limiting enzyme that regulates amyloidogenic APP processing, and the amount of the N-terminal fragment [soluble (s)APPβ] and C-terminal fragment derived from the β-cleavage of APP (βCTF, also known as C99) are commonly used to represent BACE1 activity during APP processing. We therefore assessed the levels of these two fragments, C99 and sAPPβ, in the cortex of the three groups of mice. Consistent with a change of BACE1 expression, the levels of both C99 and sAPPβ in the cortex of the 5XFAD mice that had been treated with triptolide were decreased obviously compared with those of the 5XFAD mice that had been treated with saline, whereas in the wild-type mice, sAPPβ and C99 were barely detectable (Fig. 5D,F,H). In addition, we also detected expression of the α-secretase ADAM10 and C83, a product of APP cleavage by α-secretase, in the brains of these three groups. We did not find any effect of triptolide on the ADAM10 and C83 protein levels in the brains of the 5XFAD mice, although a decrease in the protein level of ADAM10 in the cortex of 5XFAD mice was observed in comparison to that of the wild-type mice, as shown in the Fig. 5I,J. These data suggest that triptolide prevents the β-cleavage of APP.

### Treatment with triptolide exerts anti-inflammatory effects on the brains of 5XFAD mice

**Fig. 6.**
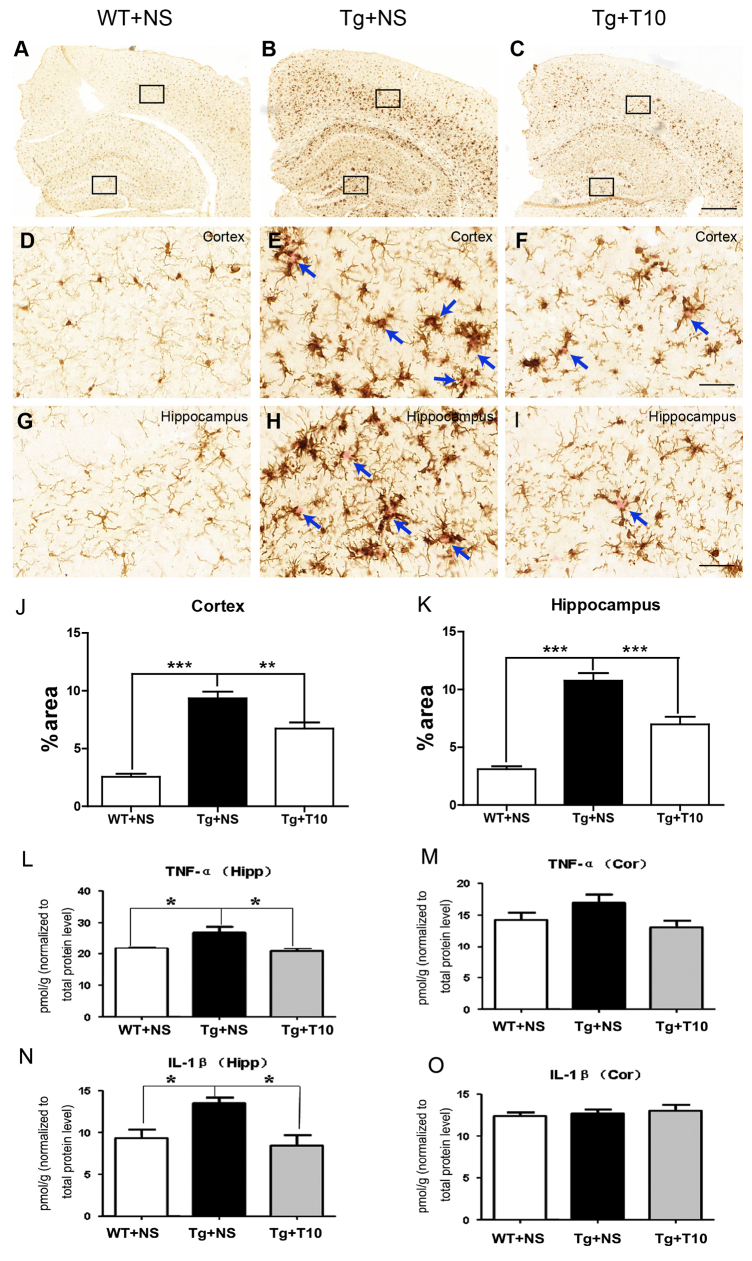
**Administration of triptolide inhibited the activation of microglia and the release of proinflammatory factors.** (A–C) Coronal sections of the three groups of mice indicated were stained for microglia with an antibody against Iba1 (immunohistochemical staining with DAB). (D–F) High magnification of Iba1 staining in the cortex area bracketed in A–C (upper box). (G–I) High magnification of Iba1 staining in the hippocampus bracketed in A–C (lower box). Arrows indicate Congo Red staining of senile neuritic plaques. The cortical (J) or hippocampal (K) area occupied by Iba1-positive microglia was quantified using Image Pro Plus 6.0 software. Meanwhile, frozen brain tissues were homogenized and ELISA analysis was used to measure the concentrations of TNFα (L,M) and IL-1β (N,O) in the cortex (Cor) and hippocampus (Hipp) of the three groups of mice. Concentrations were normalized to the brain wet weight in the hippocampus and cortex of all groups. The data are presented as means±s.e.m. *n*=6 animals per group, *P<0.05, **P<0.01, ***P<0.001 versus Tg+NS, one-way ANOVA with Tukey’s *post hoc* test. NS, normal saline; T10, triptolide. Scale bars: 500 μm (A–C); 50 μm (D–I).

Glial activation, part of an inflammatory response, is an important phenotype of AD brains (Selkoe, 2001). To determine the anti-inflammatory effect of triptolide in 5XFAD mice, the expression of the microglial marker Iba1 and the astrocyte marker GFAP was assessed by using immunohistochemical staining of brain sections from 5XFAD mice that had been treated with saline or triptolide. 5XFAD mice treated with saline exhibited microglial activation. These activated microglia were readily identified around the senile plaques, stained using Congo Red, by their thicker processes and more rounded cell bodies (Fig. 6E,H). The administration of triptolide for 8 weeks significantly inhibited activation of microglia (Fig. 6C,F,I) as more microglial cells exhibited the ramified morphology, similar to that of the wild-type mice that had been treated with saline (Fig. 6A,D,G). The area occupied by Iba1-positive microglia in the cortex and hippocampus of saline-treated 5XFAD mice was dramatically increased in association with Congo-red-stained neuritic plaques, whereas triptolide administration partially attenuated this increase (Fig. 6J,K). Compared with microglial activation, the GFAP-expressing hypertrophic astrocytes exhibited a widespread pattern in the cortex and hippocampus of 5XFAD mice treated with saline. This greater increase in GFAP-positive cells, corresponding to activated astrocytes, was found to be inhibited by triptolide administration (supplementary material Fig. S6). To quantitatively evaluate the inflammatory response in the brains of transgenic mice, we assessed the levels of the pro-inflammatory markers tumor necrosis factor α (TNFα) and interleukin-1β (IL-1β) in the cortex and hippocampus. ELISA data revealed that the concentrations of both TNFα and IL-1β were increased significantly in the hippocampus of transgenic mice, treatment with triptolide significantly attenuated this upregulation (Fig. 6L,N). However, there was no significant difference in the concentration of TNFα or IL-1β in the cortex of any of the treated groups (Fig. 6M,O), even though glial activation was observed. This suggests that the production of TNFα and IL-1β is an early and relatively transient process, rather than a prolonged and stable event, such as glial activation in the AD brain, because pathological alterations, including Aβ accumulation and glial activation, were first detected in the cortex of 5XFAD mice (Oakley et al., 2006).

### Treatment with triptolide exerts anti-oxidative effects in 5XFAD brains

**Fig. 7.**
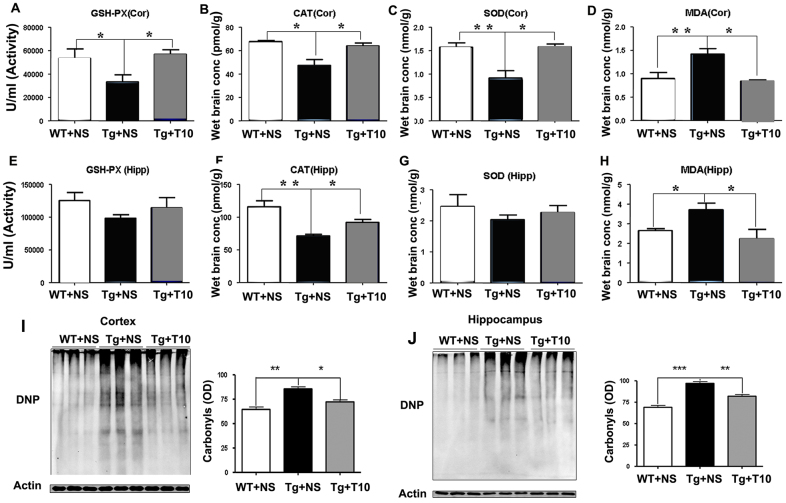
**Treatment with triptolide had anti-oxidative effects on the brains of 5XFAD mice.** (A,E) ELISA was used to detect the activity of GSH-Px in the cortex (Cor) and hippocampus (Hipp) of saline-treated wild-type mice (WT+NS), saline-treated 5XFAD mice (Tg+NS) and triptolide (T10)-treated 5XFAD mice (Tg+T10). (B,F) CAT levels in the cortex and hippocampus of the three groups of mice were measured by using ELISA. (C,G) SOD contents of the cortex and hippocampus of the three groups of mice. (D,H) The production of MDA in the cortex and hippocampus of the three groups of mice. (I,J) The protein samples of cortex or hippocampus were treated with dinitrophenyl hydrazine and subjected to western blotting. The levels of oxidative proteins were determined by using densitometry to quantify positive bands of 2,4-dinitrophenyl (DNP)-modified proteins. Actin was used as an internal control (*n*=6 animals per group), *P<0.05, **P<0.01, ***P<0.001 versus Tg+NS, one-way ANOVA with Tukey’s *post hoc* test. Conc, concentration; OD, optical density.

We next analyzed the ability of triptolide to modulate the levels and activity of antioxidant enzymes [glutathione peroxidase (GSH-Px), superoxide dismutase (SOD), catalase (CAT)] and the oxidative products of lipids and proteins [malondialdehyde (MDA) and protein carbonyl groups, respectively]. Treatment with triptolide prevented the decrease in the activity of GSH-Px, the expression of CAT and SOD in the cortex (Fig. 7A–C), and CAT levels in the hippocampus (Fig. 7F). By contrast, the activity of GSH-Px and the expression of SOD in the hippocampus were comparable in the three groups (Fig. 7E,G). In addition, triptolide reduced the production of the oxidative marker MDA in the cortex and hippocampus of 5XFAD mice by ~50% (Fig. 7D,H). Protein carbonyls, which represent the extent of protein oxidation, were upregulated in the brains of 5XFAD mice, and treatment with triptolide partially reversed these effects (Fig. 7I,J). Triptolide therefore increased the activities of anti-oxidative enzymes and subsequently reduced the production of oxidative markers in the brains of 5XFAD mice.

## DISCUSSION

The 5XFAD transgenic mouse is a powerful model to study the pathogenesis of AD and the efficacy of therapeutic agents in its treatment. The mice rapidly develop the major features of AD, including extracellular amyloid accumulation starting at 2 months of age, and memory impairment in a Y-maze test at 4 months of age (Oakley et al., 2006). To select the appropriate time point for intervention, we examined the phenotype of the 5XFAD mice and found that the deficits in spatial reference learning and memory detected by using the Morris water maze did not develop until ~4.5 months of age (Fig. 1). At this time, the accumulation of amyloid could also be detected in the CA1 and dentate gyrus of the hippocampus (supplementary material Fig. S4). Given that the Morris water maze is often used to measure spatial reference learning and memory-dependent hippocampal functions (O’Keefe and Dostrovsky, 1971; Liu and Bilkey, 2001), the accumulation of Aβ in the hippocampus is likely to play a causative role in the impairment of spatial reference learning and memory in the transgenic mice.

In the current study, we investigated the therapeutic effects of triptolide on the AD transgenic mouse model at the late disease stage. We demonstrated that triptolide improved the performance of mice learning to find the hidden platform, although it did not improve memory retention, as detected using a probe trial (Fig. 2). This could be explained by the late intervention in a mouse model with extremely rapid pathological progression. To prove this hypothesis, we brought the intervention time point forward to 3 months of age. As expected, we found that treatment with triptolide completely prevented the behavior deficits both in the hidden platform training trial and in the probe trial in the AD mice (supplementary material Fig. S7). Although the plaque number was unchanged, the mean size of Aβ-specific plaques and the area occupied by Aβ plaque load was significantly attenuated through chronic administration of triptolide, even in the dense core of the plaques in the cortex and hippocampus of 5XFAD mice (Fig. 3). Quantitative analysis by using ELISA revealed decreased levels of soluble and insoluble Aβ in the cortex and hippocampus of 5XFAD mice that had been treated with triptolide compared with that of saline-treated controls (Fig. 4). These results indicate that the Aβ burden in transgenic mouse brains was inhibited by treatment with triptolide.

Data demonstrating that treatment with triptolide did not change the expression of human APP695, but led to decreased expression of BACE1 and increased levels of NEP, in the brains of 5XFAD mice suggests that the attenuation of Aβ production by triptolide might be due to decreased Aβ production from amyloidogenic APP processing or increased Aβ clearance (Fig. 5; supplementary material Fig. S5). In addition, the products of APP processing by BACE1, the N-terminal and C-terminal fragments, sAPPβ and C99, were also reduced by triptolide treatment, suggesting that both the expression and enzymatic activity of BACE1 were downregulated after treatment with triptolide.

It is well documented that mutations in APP promote Aβ production through BACE1-mediated cleavage of APP and can therefore cause familial AD (Selkoe, 2001). However, individuals with sporadic AD can also exhibit high levels and activity of BACE1, which is correlated with increased Aβ deposition in AD brains (Yang et al., 2003; Li et al., 2004b). Recently, researchers identified a rare mutation (A673T) in APP, which appears in elderly people that are not afflicted with AD. The A673T mutation impairs BACE1-mediated cleavage of APP and is therefore protective against AD (Jonsson et al., 2012). This provides evidence that BACE1 might play a crucial role in both genetic and sporadic AD. Because of its central role in amyloidogenic processing, BACE1 could be a potential drug candidate as an early AD therapy. Given that treatment with triptolide downregulated the expression of BACE1 in the brains of 5XFAD transgenic mice (Fig. 5), it could be a promising drug candidate through acting on the early stages of AD progression by inhibiting APP processing. This inhibitory effect of triptolide on amyloidogenesis might also explain why it did not affect the performance of 5XFAD mice in a probe trial in the Morris water maze, and as such, earlier intervention might have prevented the deficits in both spatial learning and memory retention. Consistent with this hypothesis, triptolide prevented the decline in memory of transgenic mice in both escape testing and probe trials when treated at 3 months of age (supplementary material Fig. S7). It suggests that BACE1 might be the first intervention target of triptolide in the AD transgenic mouse model.

The pathogenesis of AD is complex, and as such, the inhibition of Aβ production and aggregation should not be the only intervention target. Alternatives, including anti-inflammatory and anti-oxidative drugs, have also been developed as therapeutic strategies for AD (Mangialasche et al., 2010; Ballard et al., 2011). The present study revealed that triptolide suppresses glial activation, oxidative stress and inhibits the release of pro-inflammatory factors, including TNFα and IL-1β, consistent with our previous *in vitro* findings (Jiao et al., 2008). The anti-inflammatory effects of triptolide might be due to inhibition of microglial activation through the suppression of the phosphorylation of c-jun NH_2_-terminal kinase (JNK) and the transcriptional activity of nuclear factor-κB (NF-κB) (Gong et al., 2008). It has been reported that inflammation and oxidative stress upregulate the expression of BACE1 (Wang et al., 2013a) and that the TNFα and TNF receptor 1 cascade is involved in APP processing and the formation of Aβ plaques (He et al., 2007). This evidence suggests that triptolide-mediated downregulation of BACE1 expression and the decrease in Aβ deposition in the 5XFAD mice might result from anti-inflammatory and anti-oxidative actions of triptolide (Zhou et al., 2012). However, our *in vitro* data (supplementary material Fig. S8) and observations from other laboratories (Wang et al., 2013b) revealed that triptolide inhibited Aβ production in human embryonic kidney 293 (HEK293) cells carrying the APPswe mutation (293APPswe cells, see Materials and Methods), suggesting that triptolide might also inhibit amyloidogenic APP processing in anti-inflammatory- and anti-oxidative-independent mechanisms. Furthermore, our study is the first to establish that triptolide inhibited the β-cleavage of APP in the presence of a γ secretase inhibitor in 293APPswe cell cultures (supplementary material Fig. S8). Taken together, these data suggest that the molecular mechanisms by which triptolide inhibits APP processing are mainly owing to the inhibition of BACE1 expression and activity, leading to the reduced production of Aβ from the β-cleavage of APP.

The molecular target of triptolide in the AD brain is yet to be identified. Triptolide can suppress global transcription by targeting XPB, a subunit of the basal transcription and repair factor II H (TFIIH), and inhibiting the activity of RNA polymerase-II (RNAPII) in tumor cells (Titov et al., 2011). The decreased protein levels of BACE1 in the brains of 5XFAD mice treated with triptolide might be due to the inhibitory actions of triptolide on RNAPII-mediated transcription. However, the observation that the expression of NEP is upregulated after treatment with triptolide does not support this hypothesis. Furthermore, we transfected 293APPswe cells with a luciferase-reporter plasmid driven by a 2.0 kb human *BACE1* promoter and detected the promoter activity of the *BACE1* gene. We did not find any effect of triptolide even at the final concentration of 50 nM, at which triptolide can inhibit Aβ generation from the cells (supplementary material Fig. S8G), as such an XPB- and RNAPII-mediated mechanism could not be involved in the action of triptolide. It is likely that XPB is not the molecular target of triptolide in an AD brain. Previous studies have revealed that triptolide is a specific inhibitor of the cell surface metalloproteinase ADAM10 (Soundararajan et al., 2009), which is an α-secretase for APP that can prevent the generation of Aβ and therefore limit neurodegeneration and memory loss (Pruessmeyer and Ludwig, 2009). In the present study, we did not detect a decrease in the level of ADAM10 protein and the α-cleavage of APP in 5XFAD mice that had been treated with triptolide (Fig. 5). The findings suggest that in the AD transgenic mice brain, triptolide could not inhibit ADAM10 expression and activity owing to its anti-inflammatory and anti-oxidative effects; however, the molecular mechanisms by which triptolide inhibits APP processing through anti-inflammatory and anti-oxidative pathways *in vivo* requires additional studies. Importantly, however, we observed the ability of triptolide to inhibit BACE1 expression and activity both *in vivo* and *in vitro*, as well as to rapidly cross the blood brain barrier (supplementary material Table S1). No obvious side effects of triptolide in peripheral organs were detected (supplementary material Fig. S2).

Based on this study, we propose that the improved spatial learning in 5XFAD mice after treatment with triptolide can be attributed to a decreased amyloid burden, the suppression of glial activation and reduced oxidative stress. Intriguingly, inhibition of BACE1 expression and activity appears to be a newly identified molecular mechanism of triptolide in AD brain. Triptolide (or its derivatives) could therefore be a promising therapeutic agent for the treatment of AD, especially at early stages.

## MATERIALS AND METHODS

### Animals and triptolide treatment

APP/PS1 (5XFAD) double transgenic mice co-express the human APP and PS1 transgenes (number 006554, Jackson Laboratory), and contain five familial AD mutations (APPSwFlLon, PSEN1*M146L*L286V) under the transcriptional control of the neuron-specific mouse *Thy1* promoter. We used female mice, aged between 3 and 5 months, and non-transgenic wild-type littermates served as the control group. The 5XFAD mice were originally obtained from Jackson Laboratory and were maintained by crossing heterozygous transgenic mice with C57BL/6J wild-type breeders. Genotyping was performed using PCR analysis of tail DNA. Animals were housed in cages in a controlled environment (22–25°C, 50% humidity and a 12 hour light:dark cycle) with free access to standard laboratory chow and distilled water. Five-month-old transgenic mice in the triptolide-treated group were given an intraperitoneal injection of triptolide (20 μg/kg of bodyweight), based on a previous study (Zhou et al., 2005), once every other day for 8 weeks, whereas wild-type or transgenic littermates were treated with normal saline as controls. All efforts were made to minimize animal suffering and the number of animals used. All procedures performed in this study were in accordance with the Chinese regulations involving animal protection and were approved by the animal ethics committee of the China Capital Medical University.

Triptolide was purchased from Medicass Biotechnologies (Beijing). Its molecular structure (supplementary material Fig. S1) was identified by using mass spectrometry, and the purity of the compound reached 99%, as analyzed by using reversed-phase high-performance liquid chromatography (HPLC).

### Morris water maze

The learning and memory of the mice was assessed using the Morris water maze test (Morris, 1984). The pool was arbitrarily divided into four quadrants (training, adjacent left, adjacent right and opposite). The mice were first introduced to the visible platform on day one to habituate them to the experimental environment. The platform was then moved to another quadrant and hidden beneath the surface of the water. Mice were then given three trials per day for 4 or 5 consecutive days to measure their ability to navigate to the hidden platform, beginning from separate quadrants with their heads facing the wall. Each trial ended either when an animal climbed onto the platform or when a maximum of 60 seconds had elapsed. Next, they were allowed to remain on the platform for 30 seconds. Twenty-four hours after the last training session, all mice were given a probe test for 30 seconds, where the platform was removed from the pool, and the time taken to cross to the quadrant originally containing the platform was assessed.

### Histology and immunostaining

After behavioral tests had been completed, the mice were anesthetized using 10% chloral hydrate (7.5 ml/kg of body weight, intraperitoneal), transcardially perfused with 0.9% sodium solution and decapitated. One hemi-brain of each mouse was fixed in 4% paraformaldehyde in PBS and cryoprotected in 30% sucrose solution in 0.01 M PBS. Brains were sectioned along the coronal plane on a freezing microtome at 30 μm and were allowed to air dry on SuperPlus glass slides. Standard Avidin-Biotin Complex (ABC) staining methods were then used to assess the distribution and morphology of amyloid plaques and microglia. The primary antibodies were as follows: mouse monoclonal anti-Aβ 6E10 (1:1000; SIG-39300, Covance), rabbit polyclonal anti-Iba1 (1:200; 019-19741, Wako, Osaka, Japan). The secondary antibodies were as follows: biotinylated horse anti-mouse or goat anti-rabbit IgG (1:200, Vector Laboratories). The sections stained by using the ABC method were rinsed and treated with diaminobenzidine (DAB) chromogenic substrate. After DAB chromogenic reaction, sections used for microglial morphology analysis were stained with 0.5% Congo Red solution (80% methanol and 20% glycerol) for 10 minutes at room temperature followed by differentiation of alkaline ethanol solution for 30 seconds to visualize senile plaques. Sections were air-dried, dehydrated with serial concentration of ethanol (70%, 80%, 90%, 100%), xylene and coverslipped. Images were captured under a light microscope equipped with a digital camera (Olympus). The number, area, size of Aβ-positive plaques and the area occupied by Iba1-positive microglia were quantified using Image Pro Plus 6.0 software according to our previous description (Zheng et al., 2012). Briefly, ten consecutive sections of each brain were imaged together. The areas, the average size and the total counts of Aβ-positive plaques and the area occupied by Iba1-positive staining in sections per six mouse brains of each group were measured by the software. The value was normalized to the total hippocampus or cortex area in the 100× image. For each mouse, averages were calculated across images such that each animal only contributed a single value.

### Western blotting and ELISA

The other hemi-brain from each mouse was snap-frozen for biochemical assays. For western blotting, frozen hemi-brains were homogenized in RIPA buffer (50 mM Tris, pH 7.4, 150 mM NaCl, 1% Triton-X-100, 1% sodium deoxycholate, 0.1% SDS) containing protease inhibitor cocktail (1:100, Sigma-Aldrich), and centrifuged at 12,000 *g* for 20 minutes at 4°C. The supernatants were collected, and total protein levels were quantified by using the Bradford method using a UV 1700 PharmaSpec ultraviolet spectrophotometer following the manufacturer’s instructions (Bio-Rad). Equal amounts of protein (50 μg) were separated on 10% SDS-PAGE or NuPAGE 4–12% Bis-Tris Gels (Invitrogen), and transferred to nitrocellulose membranes (Millipore). After blocking, membranes were simultaneously labeled with rabbit polyclonal anti-BACE1 (1:1000; B0681, Sigma-Aldrich), mouse monoclonal anti-β-actin (1:5000; A5316, Sigma-Aldrich), rabbit polyclonal anti-amyloid precursor protein C-terminus, which can recognize both C99 and C83 fragments and full-length APP (1:1000; A8717, Sigma-Aldrich), and rabbit polyclonal anti-NEP (1:1000; NBP1-40625, Novus International), rabbit polyclonal anti-ADAM10 (1:1000; AB19026, Millipore). Next, the primary antibody-labeled membranes were treated with IRDyeTM-800- or IRDyeTM-700-conjugated (green and red, respectively) affinity purified anti-rabbit or anti-mouse IgG secondary antibodies (1:5000; Rockland). The bands were then visualized using the LI-COR Odyssey infrared double-fluorescence imaging system (LI-COR).

Human Aβ42, Aβ40 and sAPPβ levels were assessed by using sandwich ELISA. Frozen brain tissue was centrifuged, and the soluble supernatant fractions were collected. The insoluble materials were then dissolved with lysis buffer containing 8% SDS, 8 M urea and 5 mM EDTA. The soluble and insoluble Aβ were quantified using human Aβ42 and Aβ40 ELISA kits (KHB3441 and KHB3481, respectively; Invitrogen) following the manufacturer’s instructions. The sAPPβ level in the soluble lysate of cortex was detected with a human sAPPβ ELISA assay kit (55R-27732, Fitzgerald Industries International). The absorbance was read at 450 nm using a 96-well plate reader, and Aβ, sAPPβ levels were calculated from a standard curve and normalized to the total protein levels, determined by the BCA protein assay kit (Pierce).

ELISAs were also used to assess the expression of antioxidant enzymes – GSH-Px, SOD, CAT – and a product of lipid oxidation, MDA, in the brain samples, according to the user manual of kit (Cusabio Biotech). Briefly, 100 mg of tissue was rinsed and homogenized in 1 ml of 1×PBS. After two freeze-thaw cycles to lyse cells, the supernatants were collected and assayed at an absorption wavelength of 450 nm. All values were normalized to wet brain weight.

### Measurement of oxidized proteins

The levels of oxidized proteins containing carbonyl groups were measured using an OxyBlot kit (OxyBlot Protein Oxidation Detection Kit, Millipore) following the manufacturer’s protocol. Briefly, protein samples (20 μg/lane) were treated with dinitrophenyl hydrazine, separated by using 10% SDS-PAGE and then transferred to nitrocellulose membranes. Positive bands of 2,4-dinitrophenyl (DNP)-modified proteins were then visualized using the LI-COR Odyssey imaging system and quantified by densitometry.

### Analysis of transport across the brain blood barrier

Female wild-type (C57BL/6 background) mice were used, three animals per group. Mice were intravenously administered with 450 μg/kg of body weight with triptolide or saline (0.9% w/v) as control. At 5 minutes, 10 minutes, 30 minutes or 1 hour post injection, mice were anesthetized and immediately perfused with saline. The brains were removed, weighed and homogenated with isovolumetric saline on ice. Then, 1 ml of ethyl acetate was added, and samples were vortex and centrifuged at 4°C at (13,000 *g* refrigerated centrifuge). Each supernatant was mixed with 1 ml of ethyl acetate and extracted repeatedly as mentioned above. The collected supernatant was dried in a SpeedVac. The dried samples were reconstituted in 1 ml of the mixture of methanol and water (1:1) and dried again after filtration. At last, the dried samples were reconstituted in the mixture of methanol and water (1:1) and subjected to HPLC tandem mass spectrometry (HPLC-MS/MS) shortly after vortexing.

### Generation of human BACE1 gene promoter construct and luciferase activity assay

The human *BACE1* promoter region from −2000 to −1 was amplified by PCR using the primers *Xho*I site (5′-CCGCTCGAGATTCTATTTTTCCTGTAGTTTTATT-3ʹ) and −1 *Bg1*III site (5′-GAAGATCTGGCGGCGGCTGTCAAAGCCAAAAGG-3ʹ) from the human SH-SY5Y cell genome. Nucleotides in bold represent restriction enzyme cutting sites. The PCR product was cloned in front of the firefly luciferase gene of pGL3-basic vector at *Xho*I and *Bg1*III site to generate p*BACE1*-Luci reporter plasmid. The plasmid was further confirmed by DNA sequencing.

293APPswe cells were cultured in black 96-well plates and grown to approximately 70% confluence, then transfected with 0.2 μg plasmid/100 μl per well using lipo-LTX reagent (Invitrogen) according to the manufacturer’s protocol. pCMV-Renilla luciferase (Promega) was co-transfected in order to normalize transfection efficiency. At 24 hours after transfection, 25 μl of cell medium per well was replaced by 75 μl of passive lysis buffer (Promega), and cells were incubated for 10 minutes at room temperature to allow cells to lyse. The firefly luciferase activity and *Renilla* luciferase activity assays were performed sequentially in one well with a luminometer (PerkinElmer), according to the instructions for the Dual-luciferase assay (Promega). The firefly luciferase activity was normalized to the *Renilla* luciferase activity and expressed as relative luciferase units (RLU).

### Hematoxylin and eosin staining

After transcardial perfusion with 0.9% sodium solution, peripheral tissues – including heart, liver, spleen, lung and kidney – were removed and fixed in 4% paraformaldehyde in PBS. The tissues were then embedded with mineral wax and coronally sectioned into 5-μm-thick slices and were then stained with hematoxylin and eosin. The images were captured with a Nikon optical microscope.

### Cell culture and treatments

HEK293 cells stably overexpressing human APP695 containing the Swedish APP670/671 mutation (293APPswe cells) were established as previously described (Zheng et al., 2012). The cells were maintained in Dulbecco’s minimum essential medium (DMEM; Gibco), supplemented with 10% fetal bovine serum (FBS) and G418 (Merck) at a final concentration of 800 μg/ml, at 37°C in a humidified incubator under 5% CO_2_. For experiments, the media was changed to DMEM containing 3% FBS in the presence or absence of γ-secretase inhibitor I (565750-1MGCN, Millipore) at a final concentration of 20 nM for 1 hour, and cells were then treated with different concentrations of triptolide (1, 5, 10, 50 or 100 nM) for 24 hours. The reagents were initially dissolved in DMSO and then diluted as appropriate in cell culture medium. Supernatants were analyzed by ELISA, and the cells were harvested for western blotting.

### Data analysis

All values are expressed as means±s.e.m. Statistical analysis was performed using Student’s *t*-test, or one-way ANOVA followed by Tukey’s *post hoc* test using Prism5.0 (GraphPad Software). *P* values <0.05 were considered as statistically significant.

## Supplementary Material

Supplementary Material
