# Supplementary material for: Triptolide treatment reduces Alzheimer’s disease (AD)-like pathology through inhibition of BACE1 in a transgenic mouse model of AD
Source: Dis Model Mech. 2014 Dec;7(12):1385–95. doi: 10.1242/dmm.018218 (PMC4257007; doi:10.1242/dmm.018218)
Supplement: Supplementary Material [file supp_7_12_1385__index.html]

Triptolide treatment reduces Alzheimer’s disease (AD)-like pathology through inhibition of BACE1 in a transgenic mouse model of AD — Supplementary Material 

# Triptolide treatment reduces Alzheimer’s disease (AD)-like pathology through inhibition of BACE1 in a transgenic mouse model of AD

## DMM018218 Supplementary Material

**Files in this Data Supplement:**

- **Supplementary Material**
