# Supplementary material for: Triptolide treatment reduces Alzheimer’s disease (AD)-like pathology through inhibition of BACE1 in a transgenic mouse model of AD
Source: Dis Model Mech. 2014 Dec;7(12):1385–95. doi: 10.1242/dmm.018218 (PMC4257007; doi:10.1242/dmm.018218)
Supplement: Supplementary Material [file supp_7.12.1385_DMM018218.pdf]

SUPPLEMENTAL FIGURES

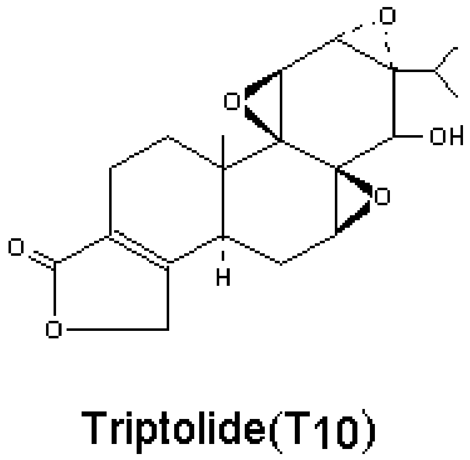

Fig. S1. The structure of triptolide. The molecular weight of the compound is 360 Da.

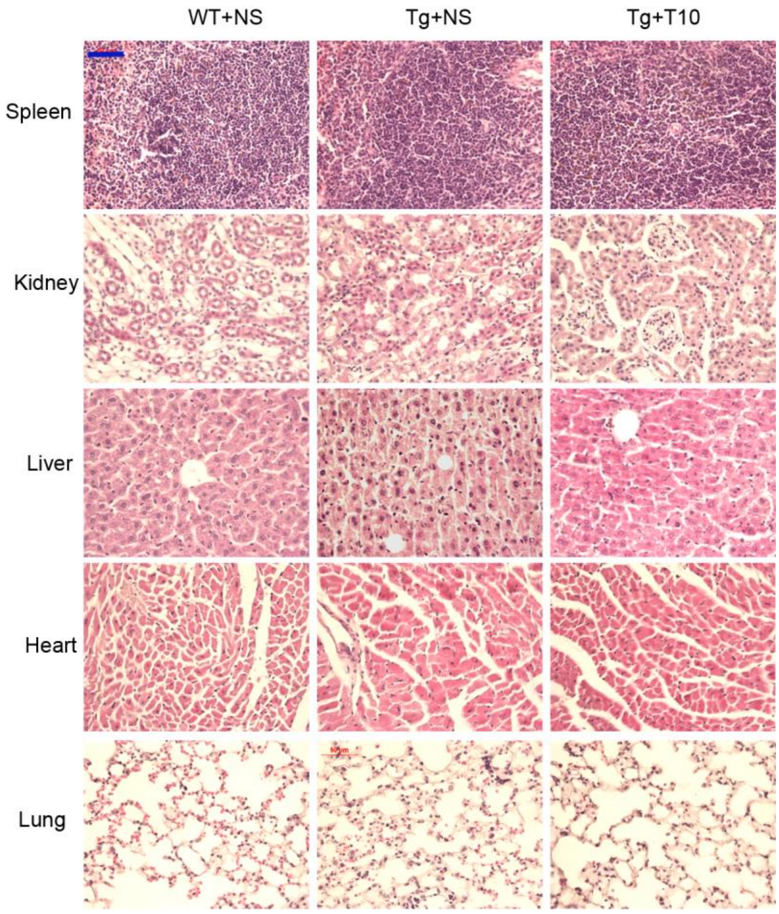

Fig. S2. Triptolide in the dose of 20 μg/kg has no toxicity in the normal or 5XFAD mice. The paraffin sections (5 μm-thickness) of peripheral tissues including spleen, kidney, liver, heart and

lung were stained with hematoxylin and eosin and images were captured by Nikon optical microscope. The scale bar is 50 μm.

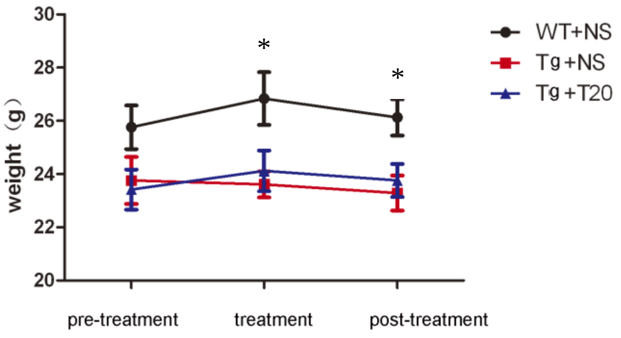

Fig. S3. Triptolide administration did not affect the body weight of 5XFAD mice. The body weight of mice in three groups were measured before, during and post treatment with triptolide, respectively. Data represent mean±SEM; n = 10 animals/group. One-way ANOVA with Tukey's post hoc analysis was used to compare these three groups, *P<0.05 vs. Tg+NS.

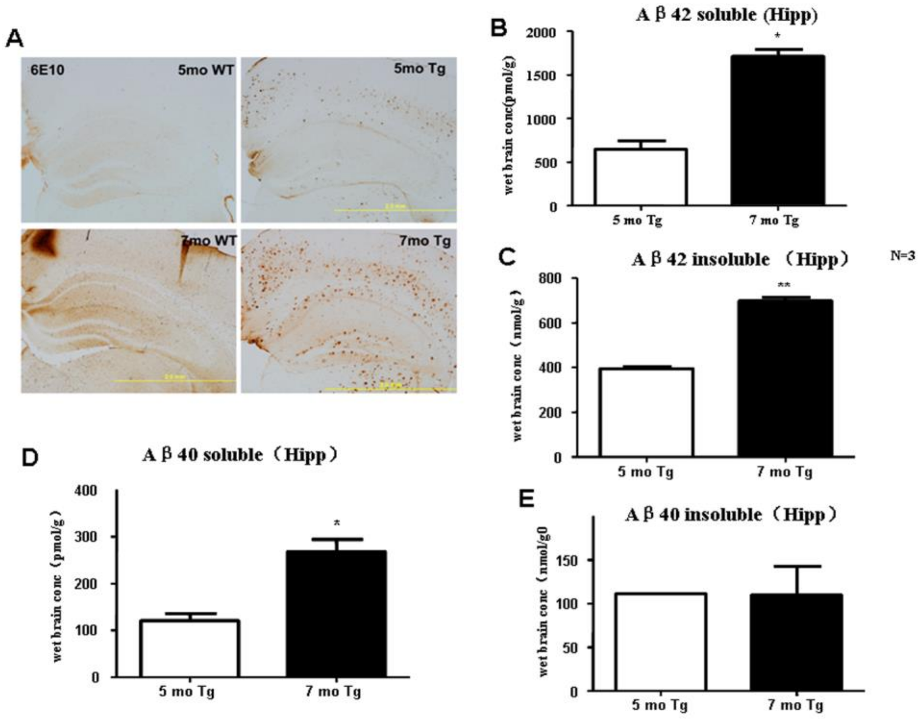

Fig. S4. The production and accumulation of Aβ increased significantly in the brains of

5XFAD mice with age. (A) Aβ-immunoreactive plaques in the brains of wild type mice and 5XFAD mice at 5 and 7 months of age, respectively. The number of Aβ-positive plaques increased in the cortex and hippocampus of 5XFAD mice with age. Scale bar = 2 mm. (B, C, D, E) ELISA results showing the production of soluble and insoluble Aβ1-42 (B, C) Levels increased with age in the hippocampus of 5XFAD mice, as did soluble Aβ1-40 (D). The increase in insoluble Aβ1-40 was not significant (E). (Mean±SEM. n=6. *P< 0.05 ** P<0.01 vs. 5XFAD at 5 months of age, Student's t test）

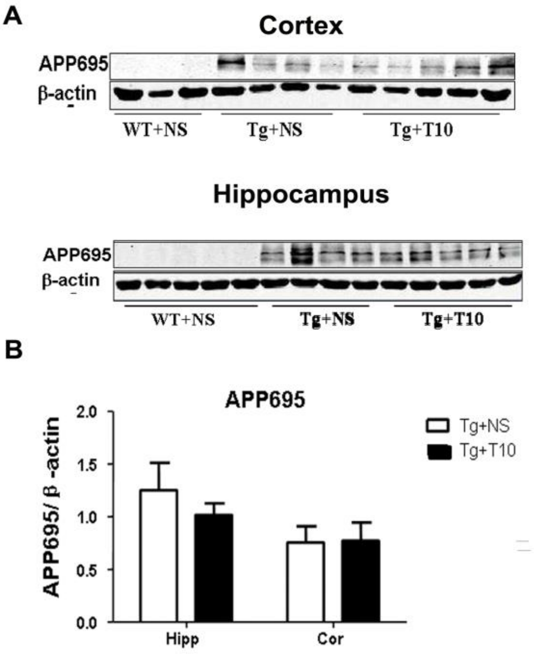

Fig. S5. Triptolide did not affect the exogenous human APP695 expression in brain of 5XFAD mice. (A) Human APP695 expression in cortex and hippocampus of these three groups of mice. Mouse monoclonal anti-APP695 antibody (1:1000, 13-0200; ZYMED Laboratories) was used to recognize the human APP695, and β-actin was used as a loading control. (B) Quantitative analysis of APP695 level normalized to β-actin.

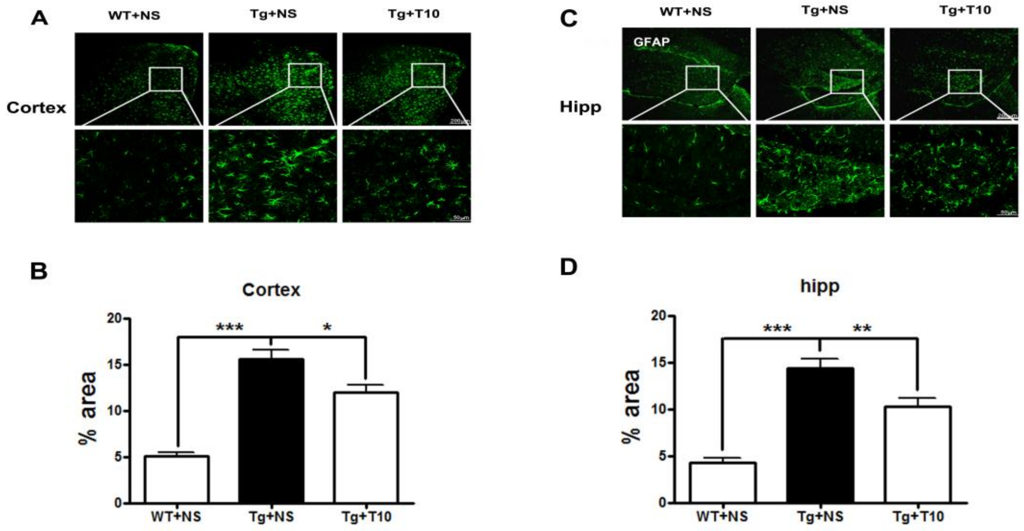

Fig. S6. Administration of triptolide alleviated the activation of astrocytes in 5XFAD mice. (A, C) Astrocytes in the cortex (A) and hippocampus (C) of the three groups of mice were detected by anti-GFAP immunofluorescent staining. (B, D) Cortical (B) or hippocampal (D) area occupied by GFAP-positive astrocyte was quantified using Image Pro Plus 6.0 software. GFAP immunoreactivity was detected using rabbit polyclonal anti-GFAP (1:500, MAB360; Millipore, Billerica, MA, USA) followed by an Alexa fluor-488 (green)-conjugated secondary antibody. The scale bar in the upper panel = 200 µm; lower panel = 50 µm. The data are presented as mean ±SEM. *n=6* animals/group; **P*<0.05; ***P*<0.01; ****P*<0.001 vs. Tg + NS, one-way ANOVA with Tukey's *post hoc* test.

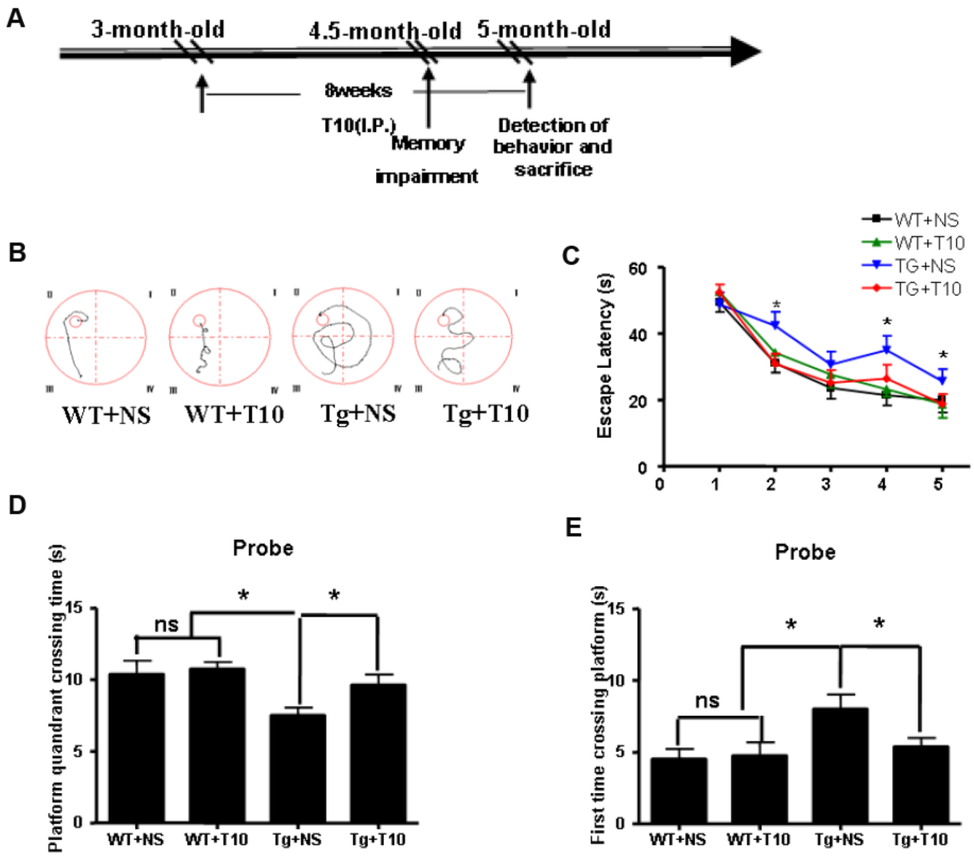

Fig. S7. Chronic administration of triptolide improved the spatial memory of 5XFAD mice at age of 3 month in the Morris water maze. (A) Treatment schedule for the experiment. (B) The escape strategy during the hidden platform task adopted by saline or triptolide (T10)-treated wild type mice (WT + NS, WT + T10), saline-treated 5XFAD mice (Tg + NS) and T10-treated 5XFAD mice (Tg + T10) was detected by camera. (C) The escape latency of these four groups of mice to find the hidden platform was recorded on every training day. Data shown as mean ±SEM; $n = 15$ animals/group. *$P < 0.05$ vs. WT + NS, one-way ANOVA with Tukey's *post hoc* test. (D, E) One day after finishing the acquisition task, a probe trial was performed to evaluate spatial memory. The platform quadrant crossing time (D), and the time required for the first crossing over the platform site (E), were used to determine the memory retention of these four groups of mice. Data shown as mean ±SEM; $n = 15$ animals/group. *$P < 0.05$ vs. Tg + NS, one-way ANOVA with

Tukey's *post hoc* test.

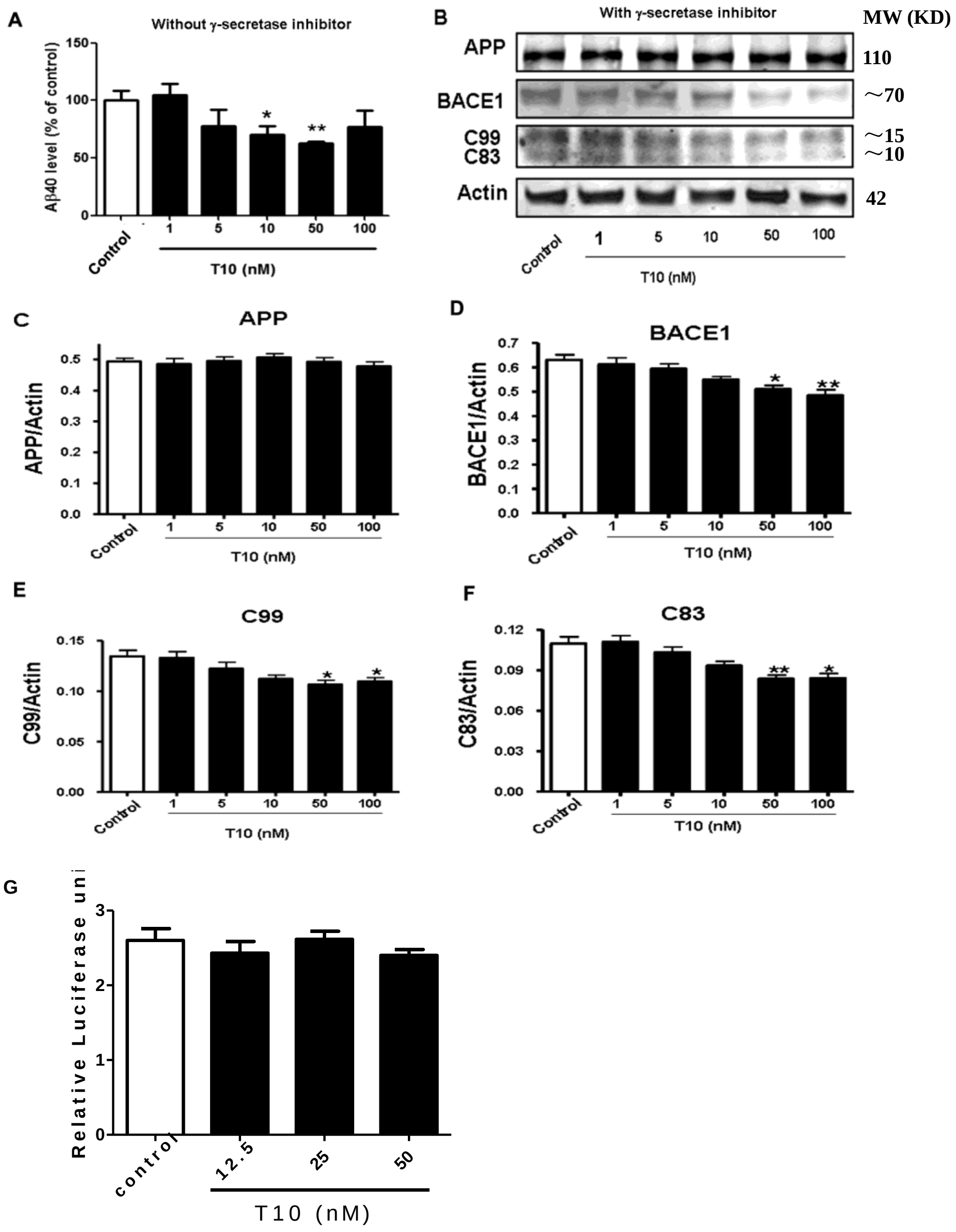

Fig. S8. Triptolide reduced the production of Aβ and amyloidogenic APP processing but did not inhibit the promoter activity of *BACE1* gene *in vitro*. (A) The 293APPswe cells were treated with different concentrations of triptolide (T10) (1, 5, 10, 50, or 100 nM) for 24 h in the absence of the γ-secretase inhibitor (20 nM). ELISA was used to detect Aβ40 secretion in

293APPswe cells. *n*=4, **P*<0.05, ***P*<0.01 vs. control. (B) In the presence of the γ-secretase inhibitor (20 nM), the levels of APP, BACE1, C99, and C83 in 293APPswe cells were determined by western blotting, and β-actin was used as a loading control. (C, D, E, F) Quantification and statistical analysis show the expression levels of APP (C), BACE1 (D), C99 (E), and C83 (F) in the cell cultures. (G) 293APPswe cells were cotranfected with Luciferase-reporter plasmid driven by a 2.01 kb human *BACE1* promoter and pCMV-Renilla-luciferase for 24 h and followed by treatment with triptolide (12.5, 25, 50 nM) or DMSO for another 12 h. The dual-luciferase activity was detected by a luminescence detector. Data are presented as mean ± SEM based on at least three independent experiments. ***P*<0.01; **P*<0.05 vs. control, one-way ANOVA with Tukey's *post hoc* test.

Table S1. Triptolide crosses the BBB to enter the brain

Time (postinjection)	Mean ± SD (ng/ml)	n
5 min	1305 ± 146.3	3
10 min	464 ± 130.8	3
30 min	81.02 ± 27.78	3
1 h	46.34 ± 28.71	3

After intravenous injection over 5 min of 450 μg/kg bw triptolide, a final concentration of triptolide at 1305 ± 146.3 ng/ml was detected in the HPLC-MS-MS assay in the brain of C57BL/6 mice (n = 3). A rapid decrease in concentration of triptolide in the brain of mice was observed in the different time points (10 min, 30 min and 1 h, respectively) postinjection.
